# Reducing excess radiation from portal imaging of pediatric brain tumors

**DOI:** 10.1120/jacmp.v14i5.4364

**Published:** 2013-09-06

**Authors:** Moses Tam, Maya Mathew, Christine J. Hitchen, Ashwatha Narayana

**Affiliations:** ^1^ Department of Radiation Oncology New York University Langone Medical Center New York; ^2^ Department of Neurosurgery New York University Langone Medical Center New York NY USA

**Keywords:** radiation therapy, pediatric brain tumors, portal imaging, IMRT

## Abstract

Previously we have shown that our routine portal imaging (PI) of the craniofacial region in pediatric brain tumor patients contributed an additional 2%‐3% of the prescribed dose and up to 200 cGy to the planning target volume (PTV) and nearby organs at risk (OARs). The purpose of this study is to quantify the reduction in dose to PTV and OARs from portal imaging (PI) of the craniofacial region of pediatric patients treated after the implementation of changes in our portal imaging practices. Twenty consecutive pediatric patients were retrospectively studied since the implementation of changes to our portal imaging procedure. Each received portal imaging of treatment fields and orthogonal setup fields to the craniofacial region. PI modifications included a reduction in the field size of setup orthogonal fields without loss of radiographic information needed for treatment verification. In addition, treatment fields were imaged using a single exposure, rather than double exposure. Dose‐volume histograms were generated to quantify the dose to the target and critical structures through PI acquisition. These results were compared with our previous cohort of 20 patients who were treated using the former portal imaging practices. The mean additional target dose from portal imaging following the new guidelines was 1.5% of the prescribed dose compared to 2.5% prior to the new portal image practices (p < 0.001). With the new portal imaging practices, the percentage decrease in portal imaging dose to the brainstem, optic structures, cochlea, hypothalamus, temporal lobes, thyroid, and eyes were 25%, 35%, 35%, 51%, 45%, 80%, and 55%, respectively. Reductions in portal imaging doses were significant in all OARs with exception of the brainstem, which showed a trend towards significance. Changes to portal imaging practices can reduce the radiation dose contribution from portal imaging to surrounding OARs by up to 80%. This may have implications on both late toxicity and second cancer development in pediatric brain tumors.

PACS number: 87

## I. INTRODUCTION

Brain tumors are the second most common cancer of childhood and account for approximately 25% of all primary pediatric tumors.^(1)^ Radiation therapy is an important treatment modality for pediatric brain tumors. However, concerns over long‐term side effects from pediatric cranial irradiation include secondary malignancies, deficits in neurocognitive and endocrine functioning, and psychosocial sequaelae.^2^, ^3^, ^4^, ^5^ Highly conformal radiation treatment plans distribute dose to the planning target volumes (PTVs) while sparing nearby organs at risk (OARs). Accurate delivery of such highly conformal dose distributions requires reproducible positioning of the patient with precise alignment of the isocenter. Portal imaging (PI) is critical to verify that the treatment isocenter and patient position match the planned isocenter and patient position.

A previous study by our institution showed that routine portal imaging of the craniofacial region in our pediatric brain tumor patients treated with intensity‐modulated radiation therapy (IMRT) contributed an additional 2%‐3% of the prescribed dose (and up to 200 cGy) to the PTVs and OARs. Hence, new practices in portal imaging were implemented for our pediatric brain tumor patients. In this study, we investigate whether these measures produce a significant reduction in dose to the PTVs and OARs.

## II. MATERIALS AND METHODS

Between April 2009 and January 2012, 20 pediatric patients (age range 1–19 years) received portal imaging of the craniofacial region during the course of radiation treatments at our institution. Twelve patients received partial brain (six brainstem and six nonbrainstem cases), six patients received craniospinal irradiation (CSI), and two patients received radiation treatment to the ventricles. All treatment were planned and delivered using 4 or 10 MV beam of Varian 2100EX linear accelerator (Varian Medical System, Palo Alto, CA). The prescription dose ranged from 3000 cGy to 5940 cGy, with a medial prescription dose of 5580 cGy. The prescribed dose per fraction was 180 cGy, with an exception of one patient who received 150 cGy per fraction. All treatment fields used intensity modulation with dynamic multileaf collimators (DMLC), with the exception that craniospinal irradiation utilized static lateral step brain fields using IMRT fields with DMLC.

**Figure 1:**
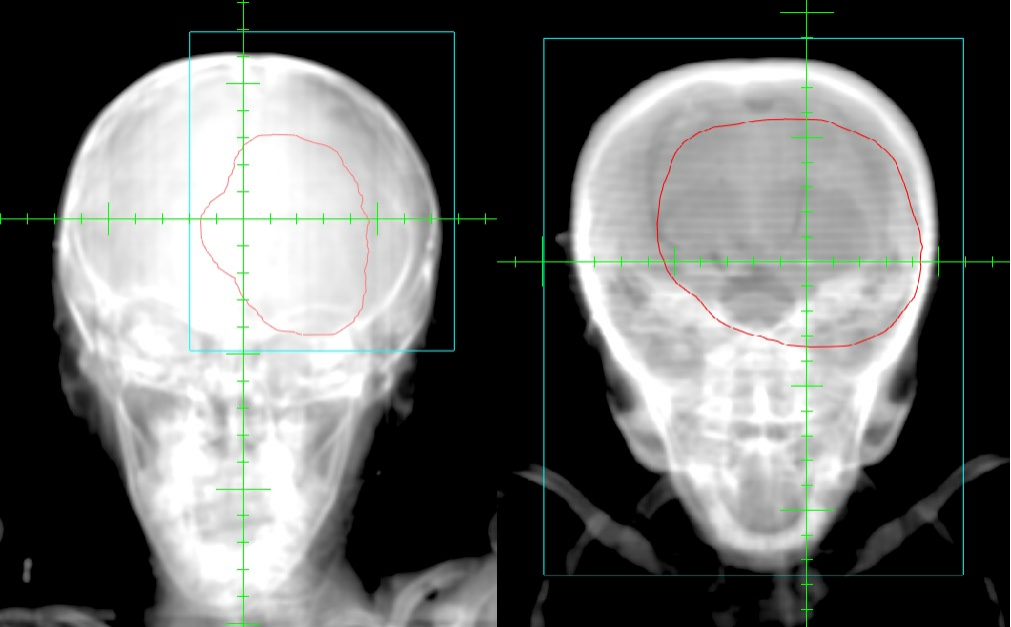
Anteroposterior setup portal image with field size delineated by blue using: (a) present portal imaging practices, and (b) former portal imaging practices.

**Figure 2:**
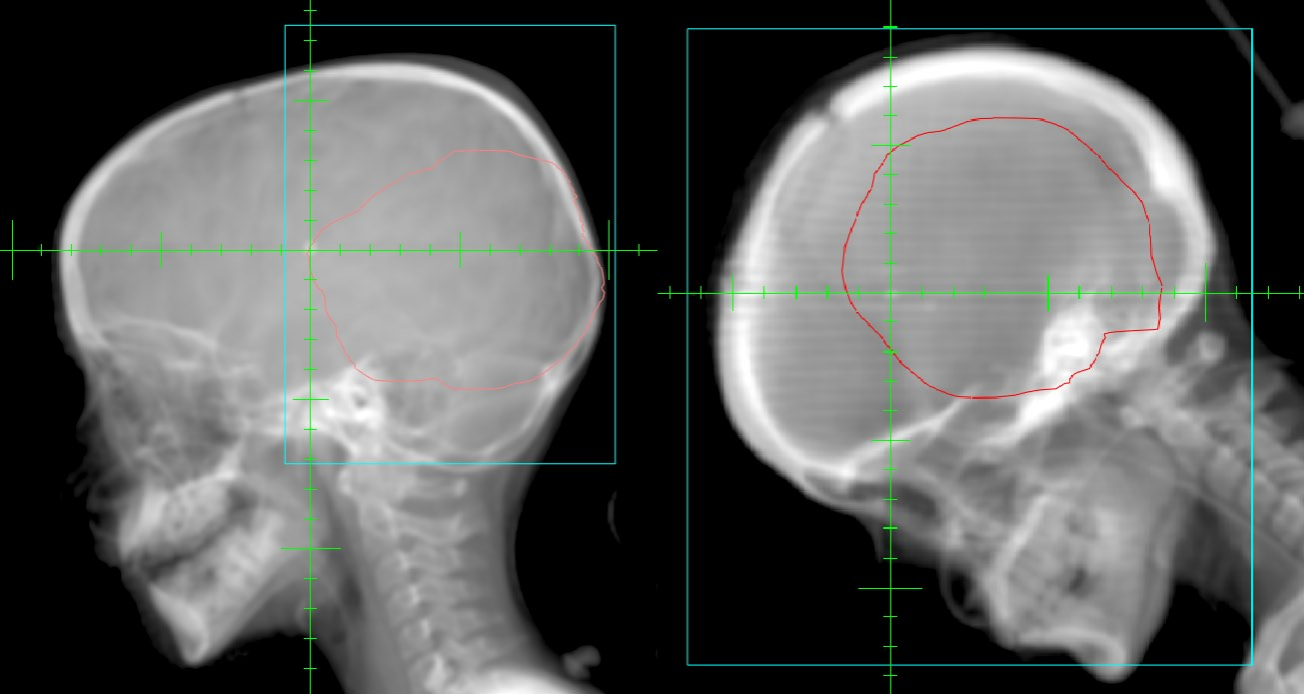
Lateral setup portal image with field size delineated by blue using: (a) present portal imaging practices, and (b) former portal imaging practices.

**Figure 3:**
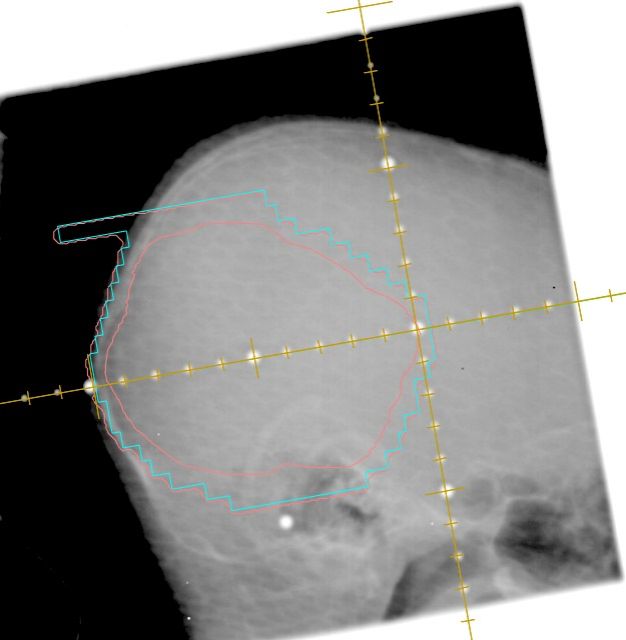
Example of a treatment field portal image. Using current practices, only a single exposure was taken of the treatment field through the completely irradiated aperture outline (CIAO), as represented by the blue line. Using former practices, an additional second exposure was taken of the treatment field plus 4 cm, as represented by the entire image.

Megavoltage (4 MV) portal images were acquired using an aS500 electronic portal imaging device (EPID) (Varian Medical System). Portal images were acquired of orthogonal setup fields for the first three days of treatment and weekly thereafter. The imaging technique for the setup fields was single exposure with limited field size to include only the surrounding anatomy necessary for isocenter and positioning verification (Figs. 1 and 2). The treatment fields were imaged on the first day of treatment with a single exposure of the completed irradiated aperture outline (CIAO) (Fig. 3).

Dose distributions and dose‐volume histograms (DVHs) were generated for PTVs and OARs for each patient using the Eclipse treatment planning system (Varian Medical System) to quantify the dose delivered through the acquisition of portal images. The dose distributions were calculated using the actual portal imaging parameters including field size, CIAO aperture, total number of monitor units (MUs), gantry angle, collimator angle, couch angle, and energy.

The doses delivered to PTVs and OARs imaged using our modified portal imaging practice were compared for this cohort of 20 patients to our previously reported cohort of 20 patients who were imaged using our former imaging procedure.

## III. RESULTS

Table 1 lists the prescription dose (median, range, dose per fraction) and the different treatment sites (brainstem, nonbrainstem, CSI, other) for the 20 patients imaged utilizing the former portal imaging practices and the 20 patients imaged using the present imaging practices.

Table 2 shows the number of portal images and the imaging monitor units for the former cohort and the present cohort. The number of PI per patient, number of PI per fraction, number of imaging monitor units per patient, and number of imaging monitor units per fraction are listed for each cohort. Using the present portal imaging practices, patients received significantly fewer portal images per fraction (p = 0.002) compared with the patients using the former imaging practices. Patients imaged with the present practices also received fewer imaging monitor units (p < 0.001) and less imaging monitor units per fraction (p = 0.001).

Table 3 demonstrates the mean maximum PI dose (expressed as cGy) to OARs for patients treated using the former portal imaging practices and the present portal imaging practices. The present portal imaging practices resulted in a significant reduction in dose delivered to the optic nerves and chiasm (35%), cochlea (35%), hypothalamus (51%), temporal lobes (45%), thyroid (80%), and eyes (55%).

Table 4 reports the mean maximum PI dose expressed as a percentage of the prescribed dose to PTVs and OARs. Using the present portal imaging practices, significantly less PI dose was delivered to the ${\text{PTV}}_{\text{mean}}$ (1.0%), ${\text{PTV}}_{\text{max}}$ (1.1%), and ${\text{PTV}}_{\text{min}}$ (0.6%), as well as to the brainstem (0.6%), optic nerves and chiasm (0.8%), cochlea (0.9%), hypothalamus (0.8%), temporal lobes (1.0%), thyroid (1.3%), and eyes (1.4%).

**Table 1:** Treatment details

	*Former PI Practice*	*Present PI Practice*
Prescription Dose		
Median (cGy)	5490	5580
Range (cGy)	4500–5940	3000–5940
Dose/fraction	180	180^a^
Treatment Sites Brainstem	7	6
Nonbrainstem	6	6
CSI^b^	4	6
Other	3	2

a Exception of 1 patient who received 150 cGy/fraction.

b CSI used static lateral step brain fields with multileaf collimators.

**Table 2:** Mean number of portal images (PI) and imaging monitor units (MUs)

	*Former PI Practice*	*Present PI Practice*	*p‐value*
No. PI/Patient	58.8	39.7	0.001
No. PI/Fraction	1.9	1.3	0.002
MU/Patient	173.3	97.9	<0.001
MU/Fraction	5.6	3.2	0.001

**Table 3:** Maximum PI dose (expressed as cGy) to OARs

	*Former PI Practice*	*Present PI Practice*	*Change (%)*	*p‐value*
OARs				
Brainstem	153	115	25	0.072
Optic nerves and chiasm	149	97	35	0.011
Cochlea	144	94	35	0.018
Hypothalamus	132	64	51	0.006
Temporal Lobes	124	69	45	<0.001
Thyroid	85	17	80	<0.001
Eyes	146	66	55	<0.001

**Table 4:** Maximum PI dose (expressed as percentage of prescribed dose) to PTV and OARs

	*Former PI Practice*	*Present PI Practice*	*Change (%)*	*p‐value*
PTV				
Mean	2.5	1.5	40	0.001
Min	2.2	1.6	28	0.009
Max	2.9	1.8	39	0.001
OARs				
Brainstem	2.8	2.2	23	0.110
Optic nerves and chiasm	2.6	1.8	30	0.021
Cochlea	2.6	1.7	32	0.029
Hypothalamus	2.4	1.6	34	0.016
Temporal Lobes	2.3	1.3	43	0.001
Thyroid	1.6	0.3	80	<0.001
Eyes	2.6	1.2	56	<0.001

## IV. DISCUSSION

Radiation therapy is an important component of multimodality therapy for childhood CNS malignancies and has contributed to the total percentage of long‐term survivors. An analysis of the Surveillance Epidemiology and End Results program showed that among survivors of childhood cancers, 24% have survived more than 30 years since their diagnosis.^(6)^ Among this group, patients with brain cancer make up the largest number of survivors. Therefore, long‐term morbidity and mortality have become increasingly important.

Our previous study revealed that radiation dose delivered from portal imaging of pediatric brain tumor patients is on average 2%‐3% of the total prescribed dose for both PTV and the surrounding OARs, which is equivalent to an additional 0.5–1.0 fraction of radiation treatment. This radiation dose is typically not considered during treatment planning or documented in the dose distributions or dose‐volume histograms. This additional dose delivered could easily exceed the tolerance limit of the surrounding OARs.^(^ ^7^ ^,^ ^8^ ^)^ Of note, the eye lens has been shown to be one of the most radiosensitive tissues in the body, and some recent studies suggest that cataract development may occur at a threshold of 0.5 Gy or even a linear, no‐threshold model.^(9)^ Our study shows that the present PI practices can significantly reduce the mean PI dose to the eye from portal imaging from 146 cGy to 66 cGy. Furthermore, the thyroid experienced the most dramatic decrease of 80% in mean PI dose, from 85 cGy to 17 cGy.

Radiation exposure contributes to an increased risk of long‐term morbidity and late mortality in patients with CNS malignancies. A study on survivors of medulloblastoma showed that both younger patients and higher doses are correlated with lower performance in neuropsychologic functioning.^(10)^ A more recent study of 1887 patients with childhood CNS tumors and a median follow up of 19.6 years showed an increased risk of developing subsequent neoplasms and neurocognitive impairment.^(5)^ The cumulative incidence of secondary neoplasm within the CNS was also associated with the maximum cranial RT dose.^(5)^ Furthermore, cranial radiation therapy dose was correlated with a decreased neurocongitive functioning in survivors of astrocytoma and glial tumors. Therefore, it may be important to reduce unnecessary radiation dose from portal imaging for pediatric brain tumors.

This study shows that new practices in the use of portal imaging can be implemented to reduce the radiation exposure that is typically unaccounted. The mean additional PI dose expressed as a percentage of the prescribed dose can be reduced by 28%‐80% dose for both PTVs and surrounding OARs. This was achieved with simple modifications to our portal imaging practices, which used smaller setup field sizes and single exposure imaging of the treatment field rather than double‐exposure imaging. It must be noted that reductions in setup field size and the use of single exposure were not performed consistently for each treatment. Therefore, additional reductions in portal imaging radiation exposure may be achieved. Additionally, the present portal imaging practices did not compromise patient care. None of these 20 pediatric patients required reimaging or a second setup.

Although this study focuses on MV imaging, the use of kilovoltage (kV) imaging is another imaging technique that would reduce the dose delivered to OARs and PTVs through portal imaging. However, kV imaging does not completely spare normal tissue to radiation and therefore the results of this paper may also be relevant.^11^, ^12^, ^13^

## V. CONCLUSIONS

Our changes in portal imaging practices reduced the radiation dose contribution from PI to surrounding OARs by up to 80%. This may have implications on both late toxicity and secondary cancer development in pediatric brain tumors.

## ACKNOWLEDGMENTS

This study did not receive any direct funding or grants.
